# Evaluation of Finnish Diabetes Risk Score in Screening Undiagnosed Diabetes and Prediabetes among U.S. Adults by Gender and Race: NHANES 1999-2010

**DOI:** 10.1371/journal.pone.0097865

**Published:** 2014-05-22

**Authors:** Lu Zhang, Zhenzhen Zhang, Yurong Zhang, Gang Hu, Liwei Chen

**Affiliations:** 1 Epidemiology Program, School of Public Health, Louisiana State University Health Sciences Center, New Orleans, Louisiana, United States of America; 2 Department of Epidemiology and Biostatistics, Michigan State University, East Lansing, Michigan, United States of America; 3 The First Affiliated Hospital of Medical School, Xi'an Jiaotong University, Xi'an, Shanxi, China; 4 Pennington Biomedical Research Center, Baton Rouge, Louisiana, United States of America; 5 Department of Public Health Sciences, Clemson University, Clemson, South Carolina, United States of America; Tulane School of Public Health and Tropical Medicine, United States of America

## Abstract

**Objective:**

To evaluate the performance of Finnish Diabetes Risk Score (FINDRISC) in detecting undiagnosed diabetes and prediabetes among U.S. adults by gender and race.

**Methods:**

This cross-sectional analysis included participants (aged ≥20 years) from the National Health and Nutrition Examination Survey (NHANES) 1999–2010. Sensitivity, specificity, area under the receiver operating characteristic (ROC) curve and the optimal cutoff points for identifying undiagnosed diabetes and prediabetes were calculated for FINDRISC by gender and race/ethnicity.

**Results:**

Among the 20,633 adults (≥20 years), 49.8% were women and 53.0% were non-Hispanic White. The prevalence of undiagnosed diabetes and prediabetes was 4.1% and 35.6%, respectively. FINDRISC was positively associated with the prevalence of diabetes (OR = 1.48 for 1 unit increase, p<0.001) and prediabetes (OR = 1.15 for 1 unit increase, p<0.001). The area under ROC for detecting undiagnosed diabetes was 0.75 for total population, 0.74 for men and 0.78 for women (p = 0.04); 0.76 for White, 0.76 for Black and 0.72 for Hispanics (p = 0.03 for White vs. Hispanics). The area under ROC for detecting prediabetes was 0.67 for total population, 0.66 for men and 0.70 for women (p<0.001); 0.68 for White, 0.67 for Black and 0.65 for Hispanics (p<0.001 for White vs. Hispanics). The optimal cutoff point was 10 (sensitivity = 0.75) for men and 12 (sensitivity = 0.72) for women for detecting undiagnosed diabetes; 9 (sensitivity = 0.61) for men and 10 (sensitivity = 0.69) for women for detecting prediabetes.

**Conclusions:**

FINDRISC is a simple and non-invasive screening tool to identify individuals at high risk for diabetes in the U.S. adults.

## Introduction

Type 2 diabetes prevalence in the U.S. is increasing dramatically, with the age-adjusted prevalence of adults (aged 18 years or older) increased from 4.5% in 1995 to 8.2% in 2010 [1]. The economic cost of diabetes in the U.S., estimated by American Diabetes Association (ADA), was $255 billion in 2012, a 41% increase since 2007 [2]. Under the current diabetes incidence trend, it is projected that 1 in every 3 U.S. adults will have diabetes (diagnosed or undiagnosed diabetes) in 2050 [3]. Additionally, a relative high rate of undiagnosed diabetes has been identified in the U.S., accounting for about 27% of the total diabetes cases of all ages [1]. Also, in 2005–2008, 35% of U.S. adults (older than 20 years) and 50% of those older than 65 years had prediabetes, a high risk stage of type 2 diabetes [4].

Emerging evidence from both observational studies and randomized controlled trials (RCTs) has clearly shown that people with high risk for type 2 diabetes or people at prediabetes stage will be benefited by early identification followed by the intensive lifestyle intervention and pharmacological treatment [5]–[9]. Thus, identifying those individuals becomes crucial and cost efficient. The traditional diabetes screening methods, including the fasting plasma glucose (FPG), the 2-hour oral glucose tolerance test (OGTT) or HbA1c test, are invasive, inconvenient and expensive [10], [11]. This is also one of the important reasons why there are a large number of diabetic patients remaining undiagnosed. Seeking a simple, reliable and cost-effective diabetes screening method, such as a diabetes risk score that can be easily conducted in clinical or community setting, has been proposed by many investigators in many countries [12]–[23]. However, a recent research indicated that diabetes risk scores derived from certain populations could have low validity when they were applied to the other populations [24]. Therefore, it is important to evaluate the performance of the diabetes risk score in a specific population before applying it in this population [24].

World-wide, more than 10 diabetes risk assessment tools have been developed from different populations [12]–[23]. Among them, Finnish Diabetes Risk Score (FINDRISC) is the most commonly used risk score in detecting undiagnosed diabetes. FINDRISC has multiple advantages over other diabetes risk scores. First, FINDRISC is a simple self-administered questionnaire which can be used as an initial diabetes screening in primary care or community settings. It can be well understood and easily calculated by the lay person or clinical personnel without any laboratory test. Second, FINDRISC was developed in a prospective study with an excellent performance in predicting the 10-year incident diabetes in Finnish population [16]. Third, FINDRISC includes 8 clearly defined questions that cover all well known risk factors of diabetes. Last but not the least, FINDRISC has been evaluated in detecting individuals with undiagnosed diabetes and prediabetes in a cross-sectional study in Finland [25] and 15 other countries or regions and gained good validity in most of these populations [24], [26]–[39]. However, most previous studies have been conducted in European countries with the majority of participants as Caucasians [26]–[32] or other single racial groups [24], [33]–[39]. The performance of the FINDRISC in the U.S., a country with multiple racial/ethnic groups, is still unknown. The aim of this study is to evaluate the performance of FINDRISC in identifying undiagnosed diabetes and prediabetes in U.S. population by sex and race/ethnicity. With the increase in the prevalence of type 2 diabetes and its associated economic burden in the U.S., identifying type 2 diabetes at early stage with simple and accurate methods becomes a public health priority.

## Methods

### Study Population (NHANES 1999–2010)

The National Health and Nutrition Examination Survey (NHANES) is designed to assess the health and nutritional status of adults and children in the U.S. through surveys including national representative sample of non-institutionalized U.S. population. From 1999, the NHANES survey became a continuous program and data were collected every 2 years. The NHANES 2009–2010 is the most updated survey released for public and research. Details of the NHANES design and procedures are available at Center for Disease Control and Prevention (CDC) website [40]. In brief, NHANES 1999–2010 includes 6 cross-sectional surveys (1999–2000, 2001–2002, 2003–2004, 2005–2006, 2007–2008, 2009–2010) that were based on a stratified multistage probability sampling design. Low-income persons, adolescents aged 12–19 years, persons aged 60 years or older, African Americans and Mexican Americans were over-sampled in NHANES surveys. Each survey included two components: a household interview and a health examination. The household interview included questionnaires on demographic, socioeconomic, dietary, and health-related information. The health examination component consisted of medical, dental, and physiological measurements, as well as laboratory tests administered by trained medical personnel in a fully equipped mobile examination center (MEC).

For this study, we included all participants from NHANES 1999–2010 aged 20 years or above who had complete information to calculate FINDRISC. The final sample size included in this analysis was 20,633.

### Finnish Diabetes Risk Score

FINDRISC was originally developed to predict the 10 years diabetes incidence in Finnish population (35 to 64 years) in a cohort study [16]. The FINDRISC is calculated based on a simple questionnaire with 8 questions, including age (years), body mass index (BMI: kg/m^2^), waist circumference (WC: cm), history of antihypertensive drug treatment, history of high blood glucose, family history of diabetes, daily consumption of fruits, berries, or vegetables (consume every day vs. not), and daily physical activity (having at least 30 minutes of physical activity during work or at leisure time vs. not) [16]. The questionnaire can be completed without any laboratory test. The answer of every question is assigned with different weighted scores according to the risk increase associated with the respective values in the regression model in the original cohort. The final score is the sum of the scores from 8 questions and ranges from 0 to 26 [25].

In our study, BMI and WC were identified from the anthropometric data measured by trained personnel in MEC. Daily physical activity time was calculated as the sum of the minutes spent on physical activity for commuting, recreation, and work on average for each day. The frequency of vegetables, fruits or berries consumption was initially collected through 24-hour food recall, and only those who consumed vegetables or fruits at least 100 grams/day were considered as consuming vegetables or fruits or berries every day. The answers to all the other questions of the FINDRISC were identified via self-reported answers from NHANES questionnaires.

### Diabetes and Prediabetes Definition and Measurement

In the present study, we categorized individuals into different groups according to their self-reported diabetes status and laboratory test results. The criteria of lab diagnosed diabetes and prediabetes were based on the most updated ADA 2013 definitions [41]. Specifically, self-reported/diagnosed diabetes was defined as having answered “Yes” to the question “Other than during pregnancy, have you ever been told by a doctor or health professional that you have diabetes or sugar diabetes?”. Undiagnosed diabetes was defined as having HbA1c ≥6.5%, or FPG ≥126 mg/dl, or 2-h OGTT plasma glucose ≥ 200 mg/dl, but not having self-reported diabetes. Diabetes included individuals either in the self-reported/diagnosed diabetes category or in the undiagnosed diabetes category. Prediabetes was defined as not having diagnosed or undiagnosed diabetes, but having HbA1c between 5.7 and 6.4%, or FPG between 100 and 125 mg/dl, or 2-h OGTT plasma glucose between 140 and 199 mg/dl. Normal glycemic level included individuals who did not have self-reported diabetes and did not meet the diabetes and prediabetes diagnosis criteria according to the FPG, 2-h OGTT, and/or HbA1c values.

### Statistical Analysis

#### Primary Analyses

Descriptive data on study participants' characteristics were expressed as means ± standard deviations (SD) for continuous variables and percentage for categorical variables. Student's t-test and χ^2^ test were applied to compare continuous and categorical variables, respectively. These procedures allow users to specify primary sampling units, stratification identification and sampling weights in the statistical procedures, as well as to generate design-adjusted means, percentages, standard errors (SE) and regression coefficients (β). Unadjusted and adjusted logistic regressions were performed to estimate the association of FINDRISC with diabetes, undiagnosed diabetes, and prediabetes, separately, where the smoking status (never, former, vs. current smoker), current alcohol drinking (yes vs. no), highest education level (with vs. without college degree or above), annual household income (more than vs. less than annual household income of $45,000) have been controlled in the adjusted analyses. Sensitivity, specificity, positive (PV+) and negative (PV-) predictive values were calculated for each FINDRISC score from 5 to 15 points for detecting undiagnosed diabetes (individuals with self-reported diabetes were excluded from this analysis) and prediabetes (individuals with self-reported or undiagnosed diabetes were excluded from this analysis). Gender- and race-specific receiver operating characteristic (ROC) curves were constructed to visually show the relationship between true-positive (sensitivity) and false positive (1-specificity). The area under the curve (AUC) was used to evaluate the performance of FINDRISC in detecting undiagnosed diabetes and prediabetes. An AUC = 0.5 indicated the test performed no better than chance and AUC = 1.0 indicated perfect discrimination. The optimal cutoff points were determined by the point with the shortest distance in the ROC curve which was calculated as the square root of [(1-sensitivity)^2^ + (1- specificity)^2^] [42]. All the statistical analyses were performed using SAS software, version 9.3 (SAS Institute, Cary, NC).

#### Secondary Analyses

Our secondary analyses were aimed to 1) compare FINDRISC with another diabetes risk score developed among Americans [12]; 2) conduct a stratified analysis by age (<65 vs. ≥65 years of age); and 3) conduct a sensitivity analysis among a subgroup of individuals who had all results of FPG, OGTT and HbA1c.

## Results

A total of 20,633 adults aged ≥20 years (mean: 47.5±17.8 years) were included in this analysis. Characteristics of participants according to the FINDRISC group were presented in Table 1. Around half of the population were women (49.79%), non-Hispanic White (52.97%), having household income less than $45,000/year (51.16%), currently married (56.42%) and having college degree or above (51.51%). The majority of the population were current alcohol drinkers (72.58%) but were non-smokers (78.47%). Among all participants of NHANES 1999–2010, the weighted percentage of self-reported/diagnosed diabetes, undiagnosed diabetes and prediabetes were 10.47%, 4.14% and 35.55%, respectively. Age, female percentage, percentage of annual household income below $45,000, percentage of married status, BMI, waist circumference, FPG, HbA1c, and systolic blood pressure increased with greater FINDRISC score (for each, P<0.0001).

**Table 1:** Characteristics of the participants aged 20 years or above in NHANES 1999–2010, by FINDRISC group.*

Characteristics	FINDRISC Group					P	All Participants
	1^st^	2^nd^	3^rd^	4^th^	5^th^		
**FINDRISC value**							
Mean (SD)	3.33 (2.05)	8.91 (1.43)	12.86 (0.83)	16.87 (1.63)	22.49 (1.38)	<.0001	9.49 (5.37)
Median (range)	4 (0–6)	9 (7–11)	13 (12–14)	17 (15–20)	22 (21–26)		9 (0–26)
**N**	6,126	7,311	3,687	2,845	664		20,633
**Age**, year	36.98 (14.28)	47.49 (17.45)	51.85 (17.17)	60.62 (13.20)	63.90 (10.11)	<.0001	47.49 (17.81)
**Female**, %	42.87	51.18	54.62	53.99	53.46	<.0001	49.79
**Race/Ethnicity**, %							
Non-Hispanic White	54.05	55.41	51.59	48.86	41.57	<.0001	52.97
Non-Hispanic Black	16.68	16.00	19.88	22.07	27.56		18.11
Hispanics	23.84	24.93	25.14	26.12	27.71		24.90
Other†	5.44	3.65	3.39	2.95	3.16		4.02
**Annual household income**, %							
Above $45,000/year	51.34	48.60	43.40	37.49	32.38	<.0001	46.42
Below $45,000/year	46.21	48.90	54.19	60.49	64.44		51.16
Other‡	2.45	2.50	2.40	2.02	3.17		2.42
**Marriage status (married)**, %	49.98	59.37	58.68	58.90	60.06	<.0001	56.42
**Education** (have college degree or above), %	57.03	52.81	49.93	41.51	37.66	<.0001	51.51
**Current smoking** (yes), %	27.39	20.76	19.27	16.03	12.20	<.0001	21.53
**Current alcohol intake** (yes), %	78.55	74.14	68.17	65.03	58.58	<.0001	72.58
**BMI**, kg/m^2^	23.68 (3.05)	28.76 (5.62)	31.47 (6.06)	32.47 (6.00)	33.65 (6.09)	<.0001	28.41 (6.18)
**Waist circumference**, cm	84.36 (8.95)	98.81 (13.16)	105.24 (13.41)	108.51 (13.10)	112.35 (13.00)	<.0001	97.44 (15.25)
**FPG** §, mg/dl	93.86 (14.40)	99.31 (21.41)	106.25 (30.00)	126.26 (51.97)	161.15 (69.65)	<.0001	104.75 (33.66)
**HbA1c**, %	5.20 (0.41)	5.41 (0.64)	5.65 (0.89)	6.30 (1.47)	7.33 (1.60)	<.0001	5.58 (0.97)
**Systolic BP**	115.81 (14.89)	122.77 (18.21)	126.46 (18.61)	132.65 (20.23)	133.39 (20.93)	<.0001	123.07 (18.70)
**Diastolic BP**	68.79 (11.55)	70.65 (13.10)	71.55 (13.59)	70.91 (14.81)	67.35 (15.20)	<.0001	70.19 (13.13)
**Diabetes**, %							
unweighted	0.93	4.17	11.09	39.30	99.10	<.0001	12.34
Weighted	1.05	4.54	11.01	39.33	98.27		10.47
**Undiagnosed diabetes**, %							
Unweighted	0.85	3.31	6.54	9.00	0.30	<.0001	3.84
Weighted	0.97	3.61	7.35	11.03	0.27		4.14
**Prediabetes**, %							
Unweighted	16.59	28.70	36.94	33.74	0.60	<.0001	26.37
Weighted	24.22	39.76	48.73	42.07	1.73		35.55

Data are means (SD) except where noted otherwise.

* FINDRISC group:  = 1 if score<7,  = 2 if score 7–11,  = 3 if score 12–14,  = 4 if score 15–20,  = 5 if score >20;

† Other race, including multiracial;

‡ The participants select the annual household income as over $20,000;

§ Fasting plasma glucose.

**Figure 1:**
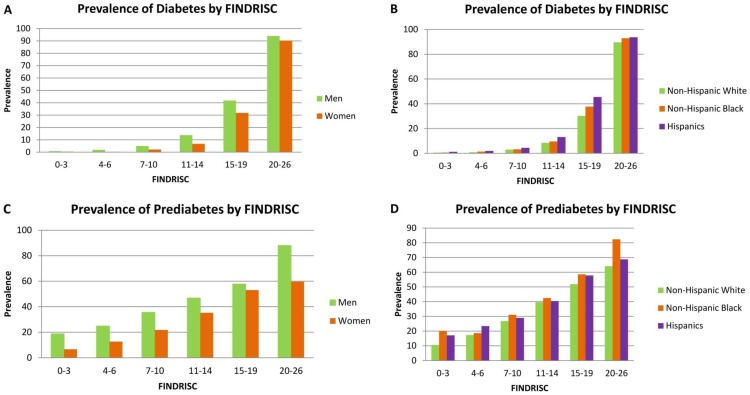
Prevalence of diabetes (A and B) and prediabetes (C and D) by Finnish Diabetes Risk Score (FINDRISC) by gender and race/ethnicity, in US men and women aged 20 years or above, NHANES 1999–2010. The prevalence of diabetes (including diagnosed and undiagnosed diabetes) was calculated among all participants; the prevalence of prediabetes was calculated among participants excluding the diagnosed and undiagnosed diabetes.

Figure 1 shows the prevalence of diabetes (both self-reported and undiagnosed diabetes, N = 20,633) and prediabetes (self-reported/diagnosed and undiagnosed diabetes excluded, N = 18,113) by gender and race/ethnicity across the six FINDRISC groups (0–3, 4–6, 7–10, 11–14, 15–19, and 20–26). The FINDRISC was positively associated with the presence of diabetes and prediabetes in all the gender and racial/ethnic groups: peaked in the highest FINDRISC score group (20–26 points). The prevalence of diabetes increased from 0.83% to 93.99% for men and from 0.44% to 90.27% for women in the lowest FINDRISC group (0–3 points) compared to the highest FINDRISC group (20–26 points) (Figure 1A). The same increasing trend was identified by racial/ethnic group. In the highest FINDRISC group (20–26 points), the prevalence of diabetes was 89.63% among non-Hispanic White, 92.95% among non-Hispanic Black and 93.77% among Hispanics (Figure 1B). The prevalence of undiagnosed diabetes (N = 18,879) was also found to be positively associated with FINDRISC after excluding the diagnosed diabetes, with 28.57% for men, 18.97% for women, 22.00% for non-Hispanic White, 29.17% for non-Hispanic Black, 15.79% for Hispanics in the highest FINDRISC group (20–26 points) (data not shown). After excluding all diabetic patients, the prevalence of prediabetes also peaked in the highest FINDRISC group (20–26 points): 88.00% for men, 59.57% for women, 64.10% for non-Hispanic White, 82.35% for non-Hispanic Black, and 68.75% for Hispanics (Figure 1 C and D). Despite of the same positive association trend for the 3 diabetic categories with FINDRISC, there were gender and racial/ethnic differences in terms of the distribution of the prevalence. Men had higher prevalence of diabetes, undiagnosed diabetes and prediabetes than women in all the FINDRISC score groups. Hispanics had a higher prevalence of diabetes while Non-Hispanic Black had a higher prevalence of undiagnosed diabetes and prediabetes over the other racial/ethnic groups.

Results from the logistic regressions also showed significant associations of FINDRISC with all diabetes, undiagnosed diabetes and prediabetes. One unit increase in FINDRISC was associated with a 48.2% (95% CI: 46.0%, 50.5%) increased odds of having diabetes, a 22.3% (95% CI: 20.0%, 24.7%) increased odds of having undiagnosed diabetes and a 14.9% (95% CI: 14.0%, 15.8%) increased odds of having prediabetes after adjusting for the smoking status, alcohol drinking, highest education level, and the annual household income (data not shown). There was no interaction between gender and FINDRISC score, indicating the associations between FINDRISC and the prevalence of diabetes, undiagnosed diabetes or prediabetes were similar comparing men with women.

As shown in Table 2, the sensitivity of FINDRISC for identifying undiagnosed diabetes and prediabetes decreased as the specificity increased in both men and women. At the cutoff value of 10 in men (sensitivity  = 74.68%; specificity = 62.74%) and 12 in women (sensitivity  = 72.17%; specificity  = 68.60%), the distance in ROC was the shortest for undiagnosed diabetes (0.45 in men and 0.42 in women). Accordingly, using the optimal cutoff point of FINDRISC of 9 in men (sensitivity  = 60.94%; specificity  = 62.43%) and 10 in women (sensitivity  = 68.72%; specificity = 60.89%) resulted in the shortest distance in ROC for prediabetes (0.54 in men and 0.50 in women). We also evaluated the racial/ethnic specific optimal cutoff points for both undiagnosed diabetes and prediabetes (data not shown). We found that the best cutoff point for undiagnosed diabetes was 11 for non-Hispanic White (sensitivity = 74.93%), 12 for non-Hispanic Black (sensitivity = 71.25%), and 11 for Hispanics (sensitivity = 65.98%). The optimal cutoff point for prediabetes was 10 for each race/ethnicity, but with the highest sensitivity for non-Hispanic Black (62.42%) and the lowest sensitivity for Hispanics (55.88%). By combining all men and women, the optimal cutoff point for undiagnosed diabetes was 11 (sensitivity = 72.13%; specificity = 65.48%); for prediabetes, the optimal cutoff point was 10 (sensitivity = 59.34%; specificity = 65.43%) in this U.S. population (data not shown).

**Table 2:** Sensitivity, specificity, positive predictive value, negative predictive value and distance in ROC of FINDRISC cutoffs in identifying undiagnosed diabetes* and prediabetes^†^ in US men and women aged 20 years or above, NHANES 1999–2010.

FINDRISC	Men						Women					
	se^‡^ %	sp^§^ %	sum∥ %	PV+¶%	PV-# %	Distance in ROC**	Se %	Sp %	Sum %	PV+ %	PV- %	Distance in ROC
Undiagnosed Diabetes												
5	93.99	26.16	120.15	6.21	98.82	0.74	98.47	16.47	114.94	4.05	99.67	0.84
6	92.92	31.77	124.69	6.62	98.85	0.69	97.55	23.66	121.21	4.38	99.63	0.76
7	90.99	38.57	129.56	7.16	98.80	0.62	96.94	28.64	125.58	4.64	99.62	0.71
8	86.27	47.66	133.93	7.90	98.52	0.54	92.97	38.45	131.42	5.13	99.35	0.62
9	83.05	54.33	137.38	8.65	98.40	0.49	91.13	45.29	136.42	5.63	99.30	0.55
10	**74.68**	**62.74**	**137.42**	**9.45**	**97.94**	**0.45**	87.77	53.31	141.08	6.31	99.19	0.48
11	66.74	69.51	136.25	10.23	97.57	0.45	79.82	61.54	141.36	6.92	98.84	0.43
12	56.44	76.06	132.50	10.93	97.11	0.50	**72.17**	**68.60**	**140.77**	**7.60**	**98.57**	**0.42**
13	46.57	82.98	129.55	12.46	96.76	0.56	63.00	77.55	140.55	9.13	98.32	0.43
14	34.98	87.99	122.97	13.17	96.30	0.66	50.46	83.09	133.55	9.65	97.91	0.52
15	15.39	91.90	107.29	14.50	96.00	0.85	41.28	88.96	130.24	11.81	97.69	0.60
Prediabetes												
5	84.60	31.86	116.46	39.71	79.59	0.70	94.74	20.32	115.06	29.02	91.82	0.80
6	81.15	38.62	119.77	41.22	79.43	0.64	91.53	28.88	120.41	30.68	90.84	0.72
7	75.70	46.14	121.84	42.71	78.16	0.59	88.79	34.64	123.43	31.84	89.98	0.66
8	67.71	55.81	123.52	44.84	76.52	0.55	81.69	45.38	127.07	33.96	87.81	0.58
9	**60.94**	**62.43**	**123.37**	**46.25**	**75.08**	**0.54**	76.77	52.87	129.64	35.90	86.87	0.53
10	52.27	70.70	122.97	48.62	73.63	0.56	**68.72**	**60.89**	**129.61**	**37.66**	**84.99**	**0.50**
11	43.96	76.65	120.61	49.96	72.06	0.61	60.80	68.22	129.02	40.45	83.70	0.50
12	35.45	82.17	117.62	51.33	70.59	0.67	52.46	75.84	128.30	42.75	82.27	0.53
13	26.75	88.14	114.89	54.46	69.40	0.74	41.34	84.05	125.39	47.12	80.64	0.61
14	19.27	91.85	111.12	55.63	68.20	0.81	32.22	88.36	120.58	48.77	79.13	0.69
15	13.73	94.89	108.62	58.76	67.46	0.86	23.02	93.08	116.10	53.37	77.86	0.77

Data are percentages (%).

* Detecting undiagnosed diabetes among all participants with complete FINDRISC information;

† Detecting prediabetes among participants with complete FINDRISC excluding undiagnosed diabetes;

‡ Sensitivity; ^§^Specificity;

∥ Sum of sensitivity and specificity;

¶ Positive predictive value;

# Negative predictive value;

** Distance in ROC curve = 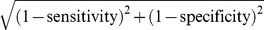.

**Figure 2:**
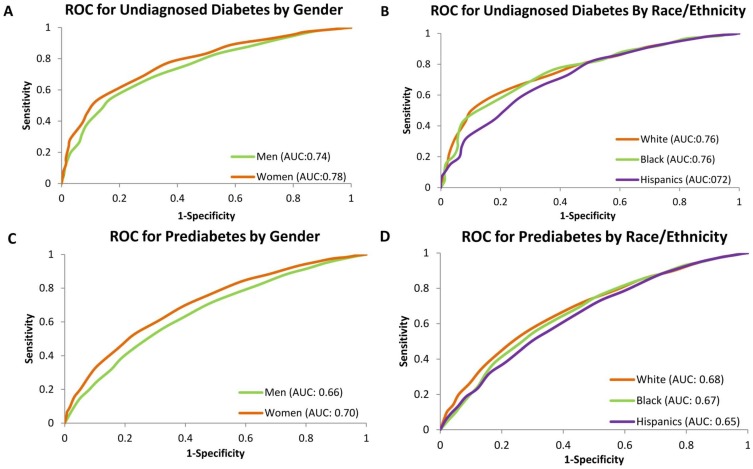
Receiver operating characteristic (ROC) curve for identifying undiagnosed diabetes (A and B) and prediabetes (C and D) by gender and race/ethnicity in US men and women aged 20 years or above, NHANES 1999–2010.

The area under the ROC curve for identifying undiagnosed diabetes were 0.74 in men and 0.78 in women (p<0.001); 0.76 for White, 0.76 for Black and 0.72 for Hispanics (p = 0.03 for White vs. Hispanics) (Figure 2 A and B). Whereas for identifying prediabetes, the area under the ROC curve was 0.66 for men and 0.70 for women (p<0.001); 0.68 for White, 0.67 for Black and 0.65 for Hispanics (p<0.001 for White vs. Hispanics) (Figure 2 C and D). After combining all the men and women participants, the area under the ROC curve was 0.75 for identifying undiagnosed diabetes and 0.67 for identifying prediabetes.

We performed stratified analysis by age (<65 vs. ≥65 years of age, men and women combined) because the original FINDRISC was developed in a population with age younger than 65 years (data not shown). For undiagnosed diabetes, the area under the ROC curve was 0.75 for all individuals, 0.75 for participants younger than 65 years (N = 17,072), and 0.65 for participants aged 65 years or above (N = 3,561). The sensitivity (cutoff point  = 10) was 75.27% for participants younger than 65 years and 87.04% for participants aged 65 years or above. For prediabetes, the area under the ROC curve was 0.67 for all individuals, 0.66 for participants younger than 65 years, and 0.57 for participants aged 65 years or above. The sensitivity (cutoff point  = 9) was 61.40% for participants younger than 65 years and 82.98% for participants aged 65 years or above.

## Discussion

In this large, cross-sectional study of national representative sample of free-living adults in the U.S., we found that the FINDRISC had good performance in identifying undiagnosed diabetes and prediabetes in both men and women in all racial/ethnic groups.

Type 2 diabetes usually starts from increased insulin resistance, a disorder that cells cannot respond to insulin normally and the pancreas gradually loses its ability to generate enough insulin. Two population-based studies have confirmed that the FINDRISC is associated with insulin resistance [43], [44], which supports the use of FINDRISC in detecting type 2 diabetes from the biological aspect.

The current ADA guideline for type 2 diabetes screening among asymptomatic population is based on the laboratory testing [45]. However, there is always a trade-off between simplicity and accuracy for each screening method. Thus, using a simple and valid questionnaire as a preliminary screening method followed with more invasive and accurate diagnosis in primary care and/or community settings, can be a cost-effective and practical method. FINDRISC is such a simple and non-invasive diabetes risk score which can be well understood by lay people and easily calculated by the lay people or clinical personnel without any laboratory test. It has been evaluated and performed well in many European countries [26]–[32]. To our knowledge, this study is the first study to validate the FINDRISC in national representative population in U.S.. As we expected, FINDRISC had a good validity in detecting undiagnosed diabetes and prediabetes in the U.S. adults. The areas under ROC curve for detecting undiagnosed diabetes were 0.74 in men and 0.78 in women in the U.S., which were very similar with those in other European populations. FINDRISC can also be used to detect prediabetes with the areas under ROC curve of 0.66 for men and 0.70 for women.

A risk score for undiagnosed diabetes using the data of NHANES 1999-2004 was developed by Bang et al. in 2009 [12]. This risk score (NHANES DRS) is calculated out of 6 variables, including age, sex, family history of diabetes, personal history of hypertension, obesity, and physical activity. Although NHANES DRS is comprised of readily available health information without using any laboratory test results and is originated among Americans, it was constructed from a crosssectional dataset. The definition of diabetes used in developing this risk score was only based on the fasting plasma glucose value, ignoring the OGTT or HbA1c value. In addition, NHANES DRS does not use any information on dietary intake which is well known to play an important role in diabetes development. However, as the NHANES DRS was developed from U.S. populations, it could be informative to compare FINDRISC and NHANES DRS using the current dataset. For undiagnosed diabetes, the area under the ROC curve of NHANES DRS was 0.77 (vs. 0.75 of FINDRISC) in all individuals, 0.76 (vs. 0.74 of FINDRISC) in men, and 0.78 in women (vs. 0.78 of FINDRISC). Although NHANES DRS has the advantage of being generated using NHANES data, the FINDRISC has very similar discriminating ability as compared to NHANES DRS from our results.

Compared with previous evaluation of FINDRISC in European populations, the most difference of this study is the composition of the study participants, the sample size and the diabetes diagnosis criteria. For every previous evaluation in Europe, the study population was all middle aged or old participants or patients with one or more cardiovascular disease risk. However, the participants of our study are nationally representative U.S. adults aged 20 years or older, which indicates the FINDRISC performs well not only in high risk population but also in the general free-living population. Although our stratified analysis indicated that the discriminating ability of FINDRISC is lower in people aged 65 years or above, the sensitivity was 87.04% for undiagnosed diabetes and 82.98% for prediabetes, which is acceptable for a first line screening tool. Thus, FINDRISC can work as an initial screening tool at the community or population level for American adults aged 20 or above, for Whites, Blacks, and Hispanics. The large sample size is also the strength of our study, which can produce more reliable results. Combined with the multiple racial/ethnic feature of our study population, this allows us to evaluate the validity of FINDRISC in different gender and racial/ethnic strata.

Using ADA diabetes diagnosis criteria 2013 to validate FINDRISC for the first time is another strength of our study. Compared with WHO diabetes diagnosis criteria or only FPG value used in previous studies of FINDRISC evaluation among other populations, our study added HbA1c as a diagnosis criterion. As a more convenient screening method than FPG and OGTT, HbA1c test does not require fasting and is not influenced by the day to day variation. HbA1c has also been found to have a very high specificity in identifying diabetic patients in different racial/ethnic groups [46], [47]. Thus, including HbA1c as diagnosis criteria for both diabetes and prediabetes leads to reducing the false negative results in our study.

Another improvement in the present study is investigating the gender and racial/ethnic difference in the performance of FINDRISC with a single population, as well as identifying the optimal cutoff point for each subgroup. Only in a cross-sectional evaluation of FINDRISC in Finland, FINDRISC was evaluated for different performance in detecting undiagnosed diabetes, abnormal glucose tolerance and metabolic syndrome by gender. FINDRISC was identified as performing a little better in women than in men only in distinguishing the metabolic syndrome without statistical significance [25]. However, in the current study among U.S. general population, FINDRISC performs significantly better in women than in men in detecting both undiagnosed diabetes and prediabetes. No previous study compared the performance of FINDRISC by race/ethnicity. In the present study we found the FINDRISC performed significantly better in non-Hispanic White than in Hispanics. The difference in the performance by gender or race/ethnicity could be due to the different sensitivity of the question by gender and race/ethnicity, such as BMI and waist circumference. One study has found that central obesity is more related with non-insulin-dependent diabetes mellitus in women than in men [48], and another study has shown that waist circumference has lower impact in metabolic syndrome in Hispanics than in non-Hispanic White and non-Hispanic Black [49]. The different performance in each subgroup even supports the rationality of evaluation of FINDRISC before applying it to a specific population. However, despite of the different performance, our results still indicate FINDRISC is a reliable screening tool in the general U.S. population. The different optimal cutoff points identified in the study could be applied to each subgroup in the practical application.

One limitation of our study is that we cannot evaluate the FINDRISC in predicting the future incident diabetes because the NHANES is a cross-sectional survey which did not provide follow-up data. However, FINDRISC was developed for both predicting future diabetes and detecting undiagnosed diabetes [16]. In addition, detecting the prediabetes in the general population also identifies the population at higher risk of type 2 diabetes in the future, given the continuum of the risk for diabetes [50]. Another limitation of current study is the possibility of misclassification on diagnosis of diabetes and prediabetes because not all participants have results from all 3 tests (i.e. FPG, OGTT, and HbA1C). To evaluate the impact of this limitation on our results, we conducted a sensitivity analysis among individuals who had results from all 3 tests (N = 3,978). The results showed that among this subgroup of individuals who had 3 tests results, the area under the ROC curve was 0.71 (vs. 0.75 for all individuals) for undiagnosed diabetes and 0.67 (vs. 0.67 for all individuals) for prediabetes, indicating the influence of the possible misclassification is minor.

Our evaluation was based on a relative healthy adult population. Different cutoff values may be applied for other populations such as children and adolescents and obese people. Further research is warrantied to investigate whether the cutoffs of FINDRISC suggested by this study could be applied to some other specific youth and obese populations given the increased prevalence of obesity and type 2 diabetes in children and adolescents in the U.S..

## Conclusions

In conclusion, FINDRISC can be used as a simple and non-invasive screening tool to identify individuals at high risk for diabetes and prediabetes in the U.S. adults. A cutoff point of 10 for men and 12 for women are suggested to identify undiagnosed diabetes; a cutoff point of 9 for men and 10 for women are suggested to identify prediabetes.

## References

[1] Centers for Disease Control and Prevention (CDC) (2012) Increasing prevalence of diagnosed diabetes - United States and puerto rico, 1995–2010. Morb Mortal Wkly Rep 61: 918–921. 23151951

[2] American Diabetes Association (2013) Economic costs of diabetes in the U.S. in 2012. Diabetes Care 36: 1033–1046. 23468086 10.2337/dc12-2625 PMC3609540

[3] Boyle JP, Thompson TJ, Gregg EW, Barker LE, Williamson DF (2010) Projection of the year 2050 burden of diabetes in the US adult population: dynamic modeling of incidence, mortality, and prediabetes prevalence. Popul Health Metr 8: 29. 20969750 10.1186/1478-7954-8-29 PMC2984379

[4] National Diabetes Fact Sheet. Available: http://www.cdc.gov/diabetes/pubs/estimates11.htm#7. Accessed 14 December 2013.

[5] Knowler WC, Barrett-Connor E, Fowler SE, Hamman RF, Lachin JM, et al (2002) Reduction in the incidence of type 2 diabetes with lifestyle intervention or metformin. N Engl J Med 346: 393–403. 11832527 10.1056/NEJMoa012512 PMC1370926

[6] Tuomilehto J, Lindstrom J, Eriksson JG, Valle TT, Hamalainen H, et al (2001) Prevention of type 2 diabetes mellitus by changes in lifestyle among subjects with impaired glucose tolerance. N Engl J Med 344: 1343–1350. 11333990 10.1056/NEJM200105033441801

[7] Vermunt PW, Milder IE, Wielaard F, de Vries JH, Baan CA, et al (2012) A lifestyle intervention to reduce Type 2 diabetes risk in Dutch primary care: 2.5-year results of a randomized controlled trial. Diabet Med 29: e223–231. 22416789 10.1111/j.1464-5491.2012.03648.x

[8] Costa B, Barrio F, Cabre JJ, Pinol JL, Cos X, et al (2012) Delaying progression to type 2 diabetes among high-risk Spanish individuals is feasible in real-life primary healthcare settings using intensive lifestyle intervention. Diabetologia 55: 1319–1328. 22322921 10.1007/s00125-012-2492-6

[9] Nilsen V, Bakke PS, Gallefoss F (2011) Effects of lifestyle intervention in persons at risk for type 2 diabetes mellitus - results from a randomised, controlled trial. BMC Public Health 11: 893. 22117618 10.1186/1471-2458-11-893 PMC3247299

[10] Icks A, Haastert B, Gandjour A, John J, Lowel H, et al (2004) Cost-effectiveness analysis of different screening procedures for type 2 diabetes: the KORA Survey 2000. Diabetes Care 27: 2120–2128. 15333472 10.2337/diacare.27.9.2120

[11] American Diabetes Association (2004) Screening for type 2 diabetes. Diabetes Care 27 Suppl 1 S11–14. 14693922 10.2337/diacare.27.2007.s11

[12] Bang H, Edwards AM, Bomback AS, Ballantyne CM, Brillon D, et al (2009) Development and validation of a patient self-assessment score for diabetes risk. Ann Intern Med 151: 775–783. 19949143 10.1059/0003-4819-151-11-200912010-00005 PMC3633111

[13] Heikes KE, Eddy DM, Arondekar B, Schlessinger L (2008) Diabetes Risk Calculator: a simple tool for detecting undiagnosed diabetes and pre-diabetes. Diabetes Care 31: 1040–1045. 18070993 10.2337/dc07-1150

[14] Hippisley-Cox J, Coupland C, Robson J, Sheikh A, Brindle P (2009) Predicting risk of type 2 diabetes in England and Wales: prospective derivation and validation of QDScore. BMJ 338: b880. 19297312 10.1136/bmj.b880 PMC2659857

[15] Kahn HS, Cheng YJ, Thompson TJ, Imperatore G, Gregg EW (2009) Two risk-scoring systems for predicting incident diabetes mellitus in U.S. adults age 45 to 64 years. Ann Intern Med 150: 741–751. 19487709 10.7326/0003-4819-150-11-200906020-00002

[16] Lindstrom J, Tuomilehto J (2003) The diabetes risk score: a practical tool to predict type 2 diabetes risk. Diabetes Care 26: 725–731. 12610029 10.2337/diacare.26.3.725

[17] Joshi SR (2005) Indian Diabetes Risk Score. J Assoc Physicians India 53: 755–757. 16334617

[18] Glumer C, Carstensen B, Sandbaek A, Lauritzen T, Jorgensen T (2004) A Danish diabetes risk score for targeted screening: the Inter99 study. Diabetes Care 27: 727–733. 14988293 10.2337/diacare.27.3.727

[19] Guasch-Ferre M, Bullo M, Costa B, Martinez-Gonzalez MA, Ibarrola-Jurado N (2012) A risk score to predict type 2 diabetes mellitus in an elderly Spanish Mediterranean population at high cardiovascular risk. PLoS One 7: e33437. 22442692 10.1371/journal.pone.0033437 PMC3307727

[20] Balkau B, Lange C, Fezeu L, Tichet J, Lauzon-Guillain BD, et al (2008) Predicting diabetes: clinical, biological, and genetic approaches - Data from the Epidemiological Study on the Insulin Resistance Syndrome (DESIR). Diabetes Care 31: 2056–2061. 18689695 10.2337/dc08-0368 PMC2551654

[21] Baan CA, Ruige JB, Stolk RP, Witteman JCM, Dekker JM, et al (1999) Performance of a predictive model to identify undiagnosed diabetes in a health care setting. Diabetes Care 22: 213–219. 10333936 10.2337/diacare.22.2.213

[22] Aekplakorn WA, Bunnag P, Woodward M, Sritara P, Cheepudomwit S, et al (2006) A risk score for predicting incident diabetes in the Thai population. Diabetes Care 29: 1872–1877. 16873795 10.2337/dc05-2141

[23] Doi Y, Ninomiya T, Hata J, Hirakawa Y, Mukai N, et al (2012) Two risk score models for predicting incident type 2 diabetes in Japan. Diabet Med 29: 107–114. 21718358 10.1111/j.1464-5491.2011.03376.x

[24] Rathmann W, Martin S, Haastert B, Icks A, Holle R (2005) Performance of screening questionnaires and risk scores for undiagnosed diabetes: the KORA Survey 2000. Arch Intern Med 165: 436–441. 15738374 10.1001/archinte.165.4.436

[25] Saaristo T, Peltonen M, Lindstrom J, Saarikoski L, Sundvall J, et al (2005) Cross-sectional evaluation of the Finnish Diabetes Risk Score: a tool to identify undetected type 2 diabetes, abnormal glucose tolerance and metabolic syndrome. Diab Vasc Dis Res 2: 67–72. 16305061 10.3132/dvdr.2005.011

[26] Franciosi M, De Berardis G, Rossi MC, Sacco M, Belfiglio M, et al (2005) Use of the diabetes risk score for opportunistic screening of undiagnosed diabetes and impaired glucose tolerance: the IGLOO (Impaired Glucose Tolerance and Long-Term Outcomes Observational) study. Diabetes Care 28: 1187–1194. 15855587 10.2337/diacare.28.5.1187

[27] Bergmann A, Li J, Wang L, Schulze J, Bornstein SR, et al (2007) A simplified Finnish diabetes risk score to predict type 2 diabetes risk and disease evolution in a German population. Horm Metab Res 39: 677–682. 17846976 10.1055/s-2007-985353

[28] Makrilakis K, Liatis S, Grammatikou S, Perrea D, Stathi C, et al (2011) Validation of the Finnish diabetes risk score (FINDRISC) questionnaire for screening for undiagnosed type 2 diabetes, dysglycaemia and the metabolic syndrome in Greece. Diabetes Metab 37: 144–151. 21144787 10.1016/j.diabet.2010.09.006

[29] Soriguer F, Valdes S, Tapia MJ, Esteva I, Ruiz de Adana MS, et al (2012) Validation of the FINDRISC (FINnish Diabetes RIsk SCore) for prediction of the risk of type 2 diabetes in a population of southern Spain. Pizarra Study. Med Clin (Barc) 138: 371–376. 21939990 10.1016/j.medcli.2011.05.025

[30] Hellgren MI, Petzold M, Bjorkelund C, Wedel H, Jansson PA, et al (2012) Feasibility of the FINDRISC questionnaire to identify individuals with impaired glucose tolerance in Swedish primary care. A cross-sectional population-based study. Diabet Med 29: 1501–1505. 22443428 10.1111/j.1464-5491.2012.03664.x

[31] Tankova T, Chakarova N, Atanassova I, Dakovska L (2011) Evaluation of the Finnish Diabetes Risk Score as a screening tool for impaired fasting glucose, impaired glucose tolerance and undetected diabetes. Diabetes Res Clin Pract 92: 46–52. 21242013 10.1016/j.diabres.2010.12.020

[32] Alssema M, Vistisen D, Heymans MW, Nijpels G, Glumer C, et al (2011) The evaluation of screening and early detection strategies for type 2 diabetes and impaired glucose tolerance (DETECT-2) update of the Finnish diabetes risk score for prediction of incident type 2 diabetes. Diabetologia 54: 1004–1012. 21153531 10.1007/s00125-010-1990-7

[33] Winkler G, Hidvegi T, Vandorfi G, Balogh S, Jermendy G (2013) Prevalence of undiagnosed abnormal glucose tolerance in adult patients cared for by general practitioners in Hungary. Results of a risk-stratified screening based on FINDRISC questionnaire. Med Sci Monit 19: 67–72. 23344680 10.12659/MSM.883747 PMC3629009

[34] Lin JW, Chang YC, Li HY, Chien YF, Wu MY, et al (2009) Cross-sectional validation of diabetes risk scores for predicting diabetes, metabolic syndrome, and chronic kidney disease in Taiwanese. Diabetes Care 32: 2294–2296. 19755627 10.2337/dc09-0694 PMC2782993

[35] Garcia-Alcala H, Genestier-Tamborero CN, Hirales-Tamez O, Salinas-Palma J, Soto-Vega E (2012) Frequency of diabetes, impaired fasting glucose, and glucose intolerance in high-risk groups identified by a FINDRISC survey in Puebla City, Mexico. Diabetes Metab Syndr Obes 5: 403–406. 23204848 10.2147/DMSO.S35545 PMC3508657

[36] Wang J, Zhang RY, Chen RP, Sun J, Yang R, et al (2013) Prevalence and risk factors for diabetic retinopathy in a high-risk Chinese population. BMC Public Health 13: 633. 23826664 10.1186/1471-2458-13-633 PMC3733656

[37] Alebiosu OC, Familoni OB, Ogunsemi OO, Raimi TH, Balogun WO, et al (2013) Community based diabetes risk assessment in Ogun state, Nigeria. Indian J Endocrinol Metab 17: 653–658. 23961481 10.4103/2230-8210.113756 PMC3743365

[38] Barengo NC, Acosta T, Arrieta A, Ricaurte C, Mayor D, et al.. (2013) Screening for people with glucose metabolism disorders within the framework of the DEMOJUAN project (DEMOnstration area for primary prevention of type 2 diabetes, JUAN Mina and Barranquilla, Colombia). Diabetes Metab Res Rev doi: 10.1002/dmrr.2462. 10.1002/dmrr.2462 23996584

[39] Ku GM, Kegels G (2013) The performance of the Finnish Diabetes Risk Score, a modified Finnish Diabetes Risk Score and a simplified Finnish Diabetes Risk Score in community-based cross-sectional screening of undiagnosed type 2 diabetes in the Philippines. Prim Care Diabetes 7: 249–259. 23953706 10.1016/j.pcd.2013.07.004

[40] National Center for Health Statistics. National health and nutrition examination survey. Centers for Disease Control and Prevention Web site: Available: http://www.cdc.gov/nchs/nhanes.htm. Accessed 14 December 2013.

[41] American Diabetes Association (2013) Diagnosis and classification of diabetes mellitus. Diabetes Care 36 Suppl 1 S67–74. 23264425 10.2337/dc13-S067 PMC3537273

[42] Perkins NJ, Schisterman EF (2006) The inconsistency of “optimal” cut-points using two ROC based criteria. Am J Epidemiol 163: 670–675. 16410346 10.1093/aje/kwj063 PMC1444894

[43] Brodovicz KG, Dekker JM, Rijkelijkhuizen JM, Rhodes T, Mari A, et al (2011) The Finnish Diabetes Risk Score is associated with insulin resistance but not reduced beta-cell function, by classical and model-based estimates. Diabet Med 28: 1078–1081. 21843304 10.1111/j.1464-5491.2011.03315.x

[44] Schwarz PEH, Li J, Reimann M, Schutte AE, Bergmann A, et al (2009) The Finnish Diabetes Risk Score is associated with insulin resistance and progression towards type 2 diabetes. J Clin Endocrinol Metab 94: 920–926. 19106274 10.1210/jc.2007-2427

[45] American Diabetes Association (2013) Standards of medical care in diabetes–2013. Diabetes Care 36 Suppl 1 S11–66. 23264422 10.2337/dc13-S011 PMC3537269

[46] Getaneh A, Andres R, Brillon DJ, Findley SE (2011) Hemoglobin A(1c) criterion for diabetes diagnosis among Hispanic and non-Hispanic populations. Endocr Pract 17: 210–217. 20841311 10.4158/EP10119.OR

[47] Bennett CM, Guo M, Dharmage SC (2007) HbA(1c) as a screening tool for detection of type 2 diabetes: a systematic review. Diabet Med 24: 333–343. 17367307 10.1111/j.1464-5491.2007.02106.x

[48] Haffner SM, Stern MP, Hazuda HP, Rosenthal M, Knapp JA, et al (1986) Role of obesity and fat distribution in non-insulin-dependent diabetes mellitus in Mexican Americans and non-Hispanic whites. Diabetes Care 9: 153–161. 3698781 10.2337/diacare.9.2.153

[49] Gurka MJ, Lilly CL, Oliver MN, Deboer MD (2013) An examination of sex and racial/ethnic differences in the metabolic syndrome among adults: a confirmatory factor anlysis and a resulting continuous severity score. Metabolism 63: 218–225. 24290837 10.1016/j.metabol.2013.10.006 PMC4071942

[50] Tabak AG, Herder C, Rathmann W, Brunner EJ, Kivimaki M (2012) Prediabetes: a high-risk state for diabetes development. Lancet 379: 2279–2290. 22683128 10.1016/S0140-6736(12)60283-9 PMC3891203

